# Complete Genome and Plasmid Sequences of 32 Salmonella enterica Strains from 30 Serovars

**DOI:** 10.1128/MRA.01232-18

**Published:** 2018-11-01

**Authors:** Kyrylo Bessonov, James A. Robertson, Janet T. Lin, Kira Liu, Simone Gurnik, Shaun A. Kernaghan, Catherine Yoshida, John H. E. Nash

**Affiliations:** a National Microbiology Laboratory, Public Health Agency of Canada, Guelph, Ontario, Canada; b National Microbiology Laboratory, Public Health Agency of Canada, Winnipeg, Manitoba, Canada; c National Microbiology Laboratory, Public Health Agency of Canada, Toronto, Ontario, Canada; Broad Institute of MIT and Harvard University

## Abstract

We report here 32 completed closed genome sequences of strains representing 30 serotypes of Salmonella. These genome sequences will provide useful references for understanding the genetic variation within Salmonella enterica serotypes, particularly as references to aid in comparative genomics studies, as well as providing information for improving *in silico* serotyping accuracy.

## ANNOUNCEMENT

Salmonella is the leading cause of bacterial gastroenteritis in North America, with more than 1.7 million cases per annum (1). Public health laboratories are replacing traditional serotyping with whole-genome sequencing (WGS) for faster and more accurate surveillance and outbreak detection (2). The adoption of short-read sequencing technology has generated large amounts of genomic information, but it is fragmented and does not represent the complete DNA sequence of an organism. High-quality genomes are of great value since the use of draft genomes in comparative genomic analyses is complicated due to the inability to distinguish between truly missing sequences and those which were not resolved during the assembly process. Much of the genomic information for Salmonella comes from highly prevalent serotypes, and there is an underrepresentation of the rarer serotypes. Tools for *in silico* serotype prediction, such as the *Salmonella In Silico* Typing Resource (SISTR) (3, 4), will benefit from this collection of high-quality reference genomes for 30 serotypes for which no closed genomes were previously available.

As of 9 September 2018, there were 634 fully closed genomes for Salmonella enterica in the NCBI genome database. Unfortunately, the large amounts of raw data available in the Sequence Read Archive (SRA) are composed primarily of Illumina short reads, which cannot readily circularize the Salmonella genome as one contiguous nucleic acid molecule. We have sequenced diverse serotypes of Salmonella using a combination of both Illumina and Oxford Nanopore platforms to produce high-quality *de novo* closed genomes for public health and comparative genomics applications. This data set represents 30 novel serotypes with 32 closed reference genomes (listed in Table 1).

**TABLE 1:** *Salmonella enterica* strains sequenced in this study, by serotype

Serotype	Isolate no.	Molecule type	Plasmid name	GenBank accession no.	Isolation source species	Isolation source details	Genome size (bp)
Berta	SA20141895	Chromosome		CP030005	Raccoon	NA^*a*^	4,725,468
	SA20141895	Plasmid	pSA20141895.1	CP030006	Raccoon	NA	67,730
Brandenburg	SA20064858	Chromosome		CP030002	Pig	Intestine	4,677,648
	SA20064858	Plasmid	pSA20064858.1	CP030003	Pig	Intestine	119,613
	SA20064858	Plasmid	pSA20064858.2	CP030004	Pig	Intestine	4,593
	SA20113174	Chromosome		CP029999	Pig	Intestine	4,724,618
	SA20113174	Plasmid	pSA20113174.1	CP030000	Pig	Intestine	102,921
	SA20113174	Plasmid	pSA20113174.2	CP030001	Pig	Intestine	4,251
Carrau	SA20041606	Chromosome		CP030236	NA	NA	4,524,637
	SA20041606	Plasmid	pSA20041606.1	CP030237	NA	NA	32,829
Concord	SA20094620	Chromosome		CP030185	NA	NA	4,854,398
	SA20094620	Plasmid	pSA20094620.1	CP030186	NA	NA	298,919
	SA20094620	Plasmid	pSA20094620.2	CP030187	NA	NA	106,569
	SA20094620	Plasmid	pSA20094620.3	CP030188	NA	NA	93,719
	SA20094620	Plasmid	pSA20094620.4	CP030189	NA	NA	5,350
Gaminara	SA20063285	Chromosome		CP030288	Lizard	Blood	4,834,965
	SA20063285	Plasmid	pSA20063285.1	CP030289	Lizard	Blood	117,908
	SA20063285	Plasmid	pSA20063285.2	CP030290	Lizard	Blood	3,587
	SA20063285	Plasmid	pSA20063285.3	CP030291	Lizard	Blood	1,526
Grumpensis	SA20083039	Chromosome		CP030223	NA	NA	4,688,830
	SA20083039	Plasmid	pSA20083039.1	CP030224	NA	NA	247,246
II 56:b:1,5	SA20053897	Chromosome		CP029995	Gecko	Feces	4,920,300
	SA20053897	Plasmid	pSA20053897.1	CP029996	Gecko	Feces	87,775
	SA20053897	Plasmid	pSA20053897.2	CP029997	Gecko	Feces	86,128
	SA20053897	Plasmid	pSA20053897.3	CP029998	Gecko	Feces	61,198
II 56:z10:e,n,x	SA20011914	Chromosome		CP029992	NA	NA	4,807,680
	SA20011914	Plasmid	pSA20011914.1	CP029993	NA	NA	4,593
	SA20011914	Plasmid	pSA20011914.2	CP029994	NA	NA	3,904
IIIa 63:g,z51:−	SA19981204	Chromosome		CP029991	NA	NA	4,598,348
IIIb 47:r:z53	SA20021456	Chromosome		CP030219	NA	NA	5,431,908
	SA20021456	Plasmid	pSA20021456.1	CP030220	NA	NA	159,279
	SA20021456	Plasmid	pSA20021456.2	CP030221	NA	NA	54,912
	SA20021456	Plasmid	pSA20021456.3	CP030222	NA	NA	54,448
IIIb 48:i:z	SA20121591	Chromosome		CP029989	Snake	Colon	5,361,355
	SA20121591	Plasmid	pSA20121591.1	CP029990	Snake	Colon	121,189
IIIb 59:z10:−	SA20051472	Chromosome		CP030026	NA	NA	6,125,373
	SA20051472	Plasmid	pSA20051472.1	CP030027	NA	NA	169,096
IIIb 60:z52:z53	SA20100201	Chromosome		CP030180	NA	NA	5,195,044
Isangi	SA20041605	Chromosome		CP030225	NA	NA	4,739,617
	SA20041605	Plasmid	pSA20041605.1	CP030226	NA	NA	5,410
	SA20041605	Plasmid	pSA20041605.2	CP030227	NA	NA	4,096
	SA20041605	Plasmid	pSA20041605.3	CP030228	NA	NA	3,428
	SA20041605	Plasmid	pSA20041605.4	CP030229	NA	NA	3,028
IV 45:g,z51:−	SA20080453	Chromosome		CP030194	NA	NA	4,651,373
	SA20080453	Plasmid	pSA20080453.1	CP030195	NA	NA	38,923
IV 53:z36,z38:−	SA20055162	Chromosome		CP030238	NA	NA	4,640,729
Kisarawe	SA20083530	Chromosome		CP030203	Lizard	Feces	5,062,813
	SA20083530	Plasmid	pSA20083530.1	CP030204	Lizard	Feces	138,648
	SA20083530	Plasmid	pSA20083530.2	CP030205	Lizard	Feces	33,467
	SA20083530	Plasmid	pSA20083530.3	CP030206	Lizard	Feces	27,709
Kottbus	SA20051528	Chromosome		CP030211	Pig	Lymph node	4,719,399
	SA20051528	Plasmid	pSA20051528.1	CP030212	Pig	Lymph node	4,081
	SA20051528	Plasmid	pSA20051528.2	CP030213	Pig	Lymph node	2,519
Litchfield	SA20052327	Chromosome		CP030202	Chicken	Ground meat	4,763,586
Livingstone	SA20101045	Chromosome		CP030233	Pig	Intestine	4,729,786
	SA20101045	Plasmid	pSA20101045.1	CP030234	Pig	Intestine	94,810
Mikawasima	SA20051401	Chromosome		CP030196	Human	Stool	4,869,528
	SA20051401	Plasmid	pSA20051401.1	CP030197	Human	Stool	141,502
	SA20051401	Plasmid	pSA20051401.2	CP030198	Human	Stool	134,274
	SA20051401	Plasmid	pSA20051401.3	CP030199	Human	Stool	2,729
	SA20051401	Plasmid	pSA20051401.4	CP030200	Human	Stool	2,174
	SA20051401	Plasmid	pSA20051401.5	CP030201	Human	Stool	1,814
Milwaukee	SA19950795	Chromosome		CP030175	NA	NA	4,822,474
	SA19950795	Plasmid	pSA19950795.1	CP030176	NA	NA	148,530
	SA19950795	Plasmid	pSA19950795.2	CP030177	NA	NA	131,435
Naestved	SA19992307	Chromosome		CP030207	Human	NA	4,844,554
	SA19992307	Plasmid	pSA19992307.1	CP030208	Human	NA	74,577
Ohio	SA20030575	Chromosome		CP030181	Pig	Liver	4,772,343
	SA20030575	Plasmid	pSA20030575.1	CP030182	Pig	Liver	224,430
	SA20030575	Plasmid	pSA20030575.2	CP030183	Pig	Feces	94,179
	SA20030575	Plasmid	pSA20030575.3	CP030184	Pig	Feces	2,318
	SA20120345	Chromosome		CP030024	Pig	Feces	4,755,436
	SA20120345	Plasmid	pSA20120345.1	CP030025	Pig	Feces	100,335
Oslo	SA20043041	Chromosome		CP030231	NA	NA	4,603,878
	SA20043041	Plasmid	pSA20043041.1	CP030232	NA	NA	87,319
Reading	SA20025921	Chromosome		CP030214	Bovine	Muscle	4,882,461
	SA20025921	Plasmid	pSA20025921.1	CP030215	Bovine	Muscle	152,311
	SA20025921	Plasmid	pSA20025921.2	CP030216	Bovine	Muscle	104,420
Rissen	SA20104250	Chromosome		CP030190	Chicken	Mixed organs	4,813,547
	SA20104250	Plasmid	pSA20104250.1	CP030191	Chicken	Mixed organs	111,887
	SA20104250	Plasmid	pSA20104250.2	P030192	Chicken	Mixed organs	4,096
	SA20104250	Plasmid	pSA20104250.3	CP030193	Chicken	Mixed organs	2,264
Telelkebir	SA20075157	Chromosome		CP030217	NA	NA	4,716,530
	SA20075157	Plasmid	pSA20075157.1	CP030218	NA	NA	97,234
Uganda	SA20031245	Chromosome		CP030235	NA	NA	4,522,338
Yoruba	SA20044414	Chromosome		CP030209	NA	Feed for fish	4,805,225
	SA20044414	Plasmid	pSA20044414.1	CP030210	NA	Feed for fish	92,624

a NA, not applicable.

Samples were grown on LB plates at 37°C, and genomic DNA was isolated using the Qiagen EZ1 DNA tissue kit on the Qiagen Advanced XL automated instrument, per the manufacturer’s protocol, using 190 μl of G2 buffer with 10 μl of proteinase K. Oxford Nanopore sequencing was performed at the National Microbiology Laboratory (NML) at Guelph (Ontario, Canada), using an Oxford Nanopore MinION sequencer with the default manufacturer protocol for rapid barcoding. Samples were prepared using either SQK-RBK001 or SQK-RBK004 rapid barcoding kits and subsequently run on a FLO-MIN106 R9.4 flow cell. Each multiplexed run produced between 4,719 and 111,488 reads per sample, with the mean read length ranging between 3,485 and 11,880 bp. Albacore v2.1.3, available from Oxford Nanopore, was used to perform demultiplexing, base calling, and quality filtering of the raw reads. Illumina sequencing was done at NML at Guelph on a MiSeq instrument (SY-410-1003; Illumina) using a MiSeq 600-cycle reagent kit v3 (MS-102-3003; Illumina) and Nextera XT DNA library preparation kit (FC-131-1031; Illumina). Each multiplexed run produced between 306,699 and 1,431,596 paired reads per sample. Hybrid *de novo* assemblies were produced without raw read filtering prior to assembly using the Unicycler pipeline v0.4.3 (5) and were manually reviewed to confirm completeness of the chromosome and any plasmids present. The predicted serotype was determined using the *Salmonella In Silico* Typing Resource (SISTR) (3, 4) to conﬁrm that the *in silico* predictions matched the phenotypic serotype determined by the NML Reference Laboratory for Salmonellosis at Guelph.

The high-quality closed reference genomes produced here will be useful for comparative genomics applications, as well as for epidemiological studies on outbreak detection and surveillance of Salmonella.

### Data availability.

The genome sequences for the 32 Salmonella isolates produced by the National Microbiology Laboratory Reference Laboratory for Salmonellosis at Guelph have been deposited in NCBI/DDBJ/ENA under BioProject no. PRJNA354244, PRJNA177577, and PRJNA177212. The GenBank accession numbers are all listed in Table 1. The Illumina and Oxford Nanopore raw sequence data in fastq and fast5 formats are also available in the Sequence Read Archive (SRA).
